# Current Trends in Blood Flow Restriction

**DOI:** 10.3389/fphys.2022.882472

**Published:** 2022-07-06

**Authors:** Molly Cuffe, Joel Novak, Adnan Saithna, H. Scott Strohmeyer, Emily Slaven

**Affiliations:** ^1^ School of Nutrition, Kinesiology, and Psychological Science, University of Central Missouri, Warrensburg, MO, United States; ^2^ Community Health Network, Physical Therapy & Rehab Department, Noblesville, IN, United States; ^3^ AZBSC Orthopedics, Scottsdale, AZ, United States; ^4^ Krannert School of Physical Therapy, University of Indianapolis, Indianapolis, IN, United States

**Keywords:** blood flow restriction, BFR training, kaatsu training, application, safety

## Abstract

**Purpose:** The purpose of the study was to explore how individuals in the United States of America applied BFR/KAATSU devices and administered BFR/KAATSU training. In addition, the study sought to examine safety topics related to BFR/KAATSU training.

**Methods:** The study was completed using survey research. Subjects were recruited through Facebook, email, and word of mouth. The survey was developed, piloted, and finally deployed March 22, 2021-April 21, 2021.

**Results:** In total, 148 consented to the research; 108 completed the survey, and of those 108, 70 indicated current use with BFR/KAATSU equipment. Professions represented included athletic training, personal training, physical therapy, and strength and conditioning. Among those currently using BFR/KAATSU training (*n* = 70), the following results were found. The most common devices used were inflatable devices (*n* = 43, 61.4%). Education completed prior to device administration was formal (*n* = 39, 55.7%) and/or self-directed (*n* = 37, 52.9%). Barriers were faced by 29 (41.4%) when trying to enact training. Techniques and parameters varied during application. Screening processes were used (*n* = 50, 71.4%) prior to training. The devices were used to determine restrictive pressure (*n* = 31, 44.3%), and a supine position was used most when determining initial restrictive pressure (*n* = 33, 47.1%). For subsequent restrictive pressure measurements, respondents repeated the same method used initially (*n* = 38, 54.3%). Workload was often defined as the length of time under tension/load (*n* = 22, 31.4%) and exercise was directly supervised (*n* = 52, 74.3%). Adverse effects included bruising, lightheadedness, and cramping (*n* = 15, 21.4%). The devices have also been applied on those with pathology (*n* = 16, 22.9%).

**Conclusion:** Those using blood flow restriction/KAATSU devices came from several professions and used an assortment of devices for BFR/KAATSU training. Individuals applied devices using a variety of parameters on populations for which efficacy has and has not been well defined.

## Introduction

Blood flow restriction (BFR) training involves the application of a device to an extremity to modify blood flow and may include brief and partial limitations in blood flow during exercise (Mouser et al., 2017; Mills et al., 2021). The pressure applied by the device is intended to limit arterial blood flow to a limb while fully restricting venous outflow in working muscles during exercise (Scott et al., 2015; Patterson et al., 2019). Devices used to alter blood flow vary in style. Patterson and Brandner (2018) identified types of devices which are commonly used to facilitate BFR training including KAATSU devices, knee wraps, inflatable devices, and the use of elastic tourniquets.

The KAATSU training device was the original blood flow training device. KAATSU training received a patent in the 1990s in the United States of America (Sato, 2005), and Yasuda et al. (2017) described KAATSU training devices as belts which facilitate blood pooling. Knee wraps have been described in the literature by authors as elastic in nature (Wilson et al., 2013; Head et al., 2015) and as wraps used for power lifting purposes (Luebbers et al., 2014; Luebbers et al., 2019). Loenneke and Pujol (2009) described the use of knee wraps as a form of practical occlusion (practical BFR). Inflatable devices are cuffs applied to the limb that can be inflated through an automatic device or handheld pump. Within the literature, terms such as a pressure cuff (Byrk et al., 2016) may be seen as opposed to inflatable devices or inflatable pumps. Tourniquets are air powered devices that apply pressure to a limb reducing or occluding circulation to a body part. The devices consist of an inflatable cuff, a unit which regulates pressure, and tubing which connects the cuff to the regulating unit (FDA, 2020).

Regardless of the style of device, the devices are applied proximally along a limb with minimal pressure to facilitate restriction (McEwen et al., 2019). Pressure affects blood flow in a nonlinear fashion within the brachial artery (Mouser et al., 2017), and superficial femoral artery (Crossley et al., 2019) and restriction pressures can be determined through a variety of means. Methods used to find restriction pressure include doppler ultrasound (Masri et al., 2016), the device itself (McEwan et al., 2019), subjective rating scales (Wilson et al., 2013), or capillary refill time (Freitas et al., 2021). Factors influencing the process of arterial blood restriction include the cuff’s construction and dimensions, the site of restriction, individual attributes, and individual physiology (McEwen et al., 2019; Patterson et al., 2019) such as limb circumference (Loenneke et al., 2012; Jessee et al., 2016; Sieljacks et al., 2018) and diastolic blood pressure (Loenneke et al., 2012; Sieljacks et al., 2018).

Once the device has been applied, BFR/KAATSU training can be used in conjunction with a variety of exercise techniques. Methods of exercise used with BFR/KAATSU training devices include aerobic exercise (Pattterson & Brandner, 2018; Patterson et al., 2019; Formiga et al., 2020) and resistance exercise (Hughes et al., 2017; Wilk et al., 2018; Patterson et al., 2019). One recommendation for walking or cycling with BFR has been established by Patterson et al. (2019) and includes exercising two to three times per week at less than 50% heart rate reserve, VO_2_ Max, for 5–20 min at 40–80% arterial occlusion pressure. Implementation of BFR with aerobic exercise in populations across the lifespan yielded improvements in function (Paton et al., 2017; Chen et al., 2019; Formiga et al., 2020). The use of low load resistance exercise with BFR to gain muscle strength and hypertrophy has just one of the following suggestions for use: two-four times per week using 75 repetitions (30-15-15-15) or repetitions to failure (Patterson et al., 2019). When applied with resistance training, BFR in conjunction with low load exercise was also effective at improving muscle strength and hypertrophy (Pearson & Hussain, 2015; Cook et al., 2017; Lixandrão et al., 2018).

Currently, little is known regarding how individuals are using different types of BFR/KAATSU training devices in the United States of America. The authors of three observational studies looked at experiences with BFR/KAATSU training (Nakajima et al., 2006; Yasuda et al., 2017; Patterson & Brandner, 2018). Patterson and Brandner (2018) assessed the use of BFR training globally by physicians, strength and conditioning specialists, rehabilitation specialists, sport specific scientists, personal trainers, and researchers. Authors of the remaining studies focused on the use and safety related to the KAATSU training (Nakajima et al., 2006; Yasuda et al., 2017). This study adds to the existing body of literature through its exploration of how BFR/KAATSU was being administered. Understanding how different forms of BFR/KAATSU training devices were being used can expose gaps in the literature needing further exploration. In addition, information concerning adverse effects could facilitate additional precautions when using different devices for BFR/KAATSU training. Therefore, the purpose of this study was to explore how individuals across different professions administered and used various forms of BFR/KAATSU training devices in the United States of America. In addition, the study sought to explore safety topics related to BFR/KAATSU training with various devices.

## Materials and Methods

The survey-based research study took place March 22, 2021-April 21, 2021. Prior to starting participant recruitment, the study was approved through the appropriate Institutional Review Board.

### Participants

Those using BFR/KAATSU training devices were included in the study. To be included in the study, participants met the following criteria: 1) English speaking, 2) older than 18 years old, and 3) use BFR/KAATSU training for aerobic exercise, strength training exercise, or rehabilitation purposes in the United States. Subjects were excluded if 1) BFR/KAATSU training was not being used with patients/clients/athletes.

### Data Collection

Data collection was completed in Qualtrics (Version XM), exported into Microsoft Excel (Version 2101) then the statistical software management system, IBM SPSS Statistics for Windows (Version 27).

### Instrumentation

The survey was developed following a review of previous survey-based literature (Nakajima et al., 2006; Yasuda et al., 2017; Patterson & Brandner, 2018). Conversation among research team led to the development of topic areas, and the subsequent research questions were developed by one research member. Remaining research team members and an additional external contact reviewed questions for clarity and ease of read. A test pilot of the survey was administered in November 2020. A content expert recruited subjects and served as a liaison between the researcher and the subjects taking the pilot survey to ensure anonymity. The survey was restrictively administered to a group of 10 subjects on two separate occasions, one week apart. All 10 participants of the test pilot completed the survey the first time while eight participants completed the test pilot survey the second time. Data was analyzed using IBM SPSS Statistics for Windows (Version 24). Participants from the test pilot were all Caucasian with 60% of subjects identifying as male and 40% identifying as females. All were from the Midwest with a mean age between 31 and 40 years. Participants from the test pilot represented the professions of athletic training, physical therapy, and strength and conditioning with an average time in their respective fields of less than 10 years.

The survey test pilot took participants approximately 13 min to complete. The purpose of the test pilot was to assess the content presented in the survey. Normality of the data was assessed using the Shapiro Wilks test. Subsequent Pearson correlation and Spearman Rho correlation showed significance between measures with an alpha value of *p* < 0.05. Constructs with correlations display moderate correlation to high correlation. Cronbach’s alpha on 24 applicable items was ɑ = 0.484. Considering the statistical results in conjunction with subject feedback, fifteen questions were modified or deleted. The final survey contained 37 questions.

### Procedures

Recruitment was completed through convenience and snowball sampling through Facebook and email. The following groups agreed to be a part of sampling on Facebook: Kansas City Athletic Trainers Society; Women in Athletic Training Group; and the following National Strength and Conditioning Association (NSCA) Special Interest Groups: College Coaches, Personal Trainers, Sport Science and Performance Technology, and Sports Medicine/Rehabilitation. The following groups agreed to be surveyed through email: Collegiate Strength and Conditioning Association.

The survey was available for four weeks. All subjects completed the same survey, which was developed, housed, and deployed through Qualtrics. Participants were asked up to but no more than 37 questions divided into the following sections: Informed Consent, Product Use, Current Use, Safety, Demographics of patients, clients, and athletes, and Demographics of the respondent. The Informed Consent portion of the survey housed the informed consent documentation and asked participants to consent to the research. The questions within Product Use focused on the types of BFR/KAATSU training devices both previously and currently being used by the subject. The Current Use section asked questions pertaining to the methods used to apply BFR/KAATSU training. The Safety section assessed safety related concerns and adverse effects seen when using BFR/KAATSU training devices. The final two sets of questions asked about demographics of the patients/clients/athletes for which BFR/KAATSU training was applied and the demographics of the individual completing the survey. A subject could terminate participation in the survey at any given time by closing out of the survey. At the conclusion of the survey, participants were offered the opportunity to enroll for a chance to win one of five $10 gift cards.

## Results

### Study Response Rates

The survey yielded 149 responses; 148 individuals consented to participate in the survey research. Of those consenting to the survey research, there were 40 (27%) individuals who did not complete the survey, 38 (25.7%) who were not currently using BFR/KAATSU training, and 70 (47.3%) who at the time of the survey were using BFR/KAATSU training.

### Previous BFR/KAATSU Training Use

Information regarding those previously using BFR/KAATSU training devices (*n* = 108) and those currently using BFR/KAATSU training devices (*n* = 70) can be found in Table 1. Individuals who were not actively administering BFR/KAATSU training (*n* = 38, 35.2%) were henceforth excluded. Reasons identified for no longer using BFR/KAATSU training were as follows: “I previously utilized for injury rehabilitation, is no longer necessary”, “not allowed per company because I have not taken company’s training”, “I am at a different school where we do not have blood flow restriction devices”, and “I do not have the resources in my athletic training room to use this form of rehab”.

**TABLE 1:** Previously and currently used devices as indicated by respondents.

Type of Device	Previously Used	Currently Used	Currently Used Devices Identified
Elastic tourniquet device	*n* = 21	*n* = 9	3M (*n* = 1) BFR Bands (*n* = 1) Generic brand (*n* = 1) HMKL (*n* = 1) Koala Bands (*n* = 1) Konmed/OBM (*n* = 1) Defi PTS-PBFR (*n* = 1)
Inflatable device	*n* = 47	*n* = 43	Air Bands (*n* = 4) Mad-Up (*n* = 2) Occlusion Cuffs (*n* = 1) Edge Rehab Cuffs (*n* = 2) Smart Cuffs (n = 10) B Strong (*n* = 7) Defi PTS-PBFR (*n* = 16) Fitcuffs (n = 2) H + Cuffs (n = 2) BFR Signature Series (*n* = 1) BFR Occlude (*n* = 1) Throwraft original TD 2401 (Note: this is a personal floatation device) (*n* = 1) VALD (n = 2) Unknown name (*n* = 2)
KAATSU training device	*n* = 11	*n* = 9	Air Cuffs (*n* = 1) Dumbbell pressure exercise (*n* = 1) Inflatable Cuffs (*n* = 1) KAASTU Cycle Pro (*n* = 1) Nano (*n* = 2)
Knee wraps	*n* = 11	*n* = 2	LP Sports Protector (*n* = 1)
Other	*n* = 8 BFR Bands (*n* = 1) KELVI BFR (*n* = 1) RockCuff (*n* = 1) DELFI-PTS-PBFR (*n* = 5)	*n* = 7	Ace bandages (*n* = 1) Delfi PTS-PBFR (*n* = 3) KELVI (*n* = 1) Rock Cuff (*n* = 1)

### Current BFR/KAATSU Training Use

The remaining respondents (*n* = 70) identified themselves as males (*n* = 41, 58.6%) and females (*n* = 29, 41.4%). Additional information on demographics and professional careers can be found in Table 2.

**TABLE 2:** Demographics.

Demographics of Respondents	
Gender	Male (*n* = 41) Female (*n* = 29)
Age (in years)	18–30 (*n* = 36) 31–40 (*n* = 27) 41–50 (*n* = 5) 51–60 (*n* = 1) 61 and older (*n* = 1)
Ethnicity	White (*n* = 57) Black, African American (*n* = 3) Asian (n = 1) White/Black, African American (n = 1) American Indian or Alaskan Native (*n* = 4) Asian/Native Hawaiian or Pacific Islander (*n* = 1) Hispanic or Latino/a (*n* = 1) White/Hispanic or Latino/a (*n* = 1)
	All race (*n* = 1)
Location	Northeast (*n* = 11) Southeast (n = 17) Midwest (*n* = 27) West (*n* = 7) Southwest (*n* = 7) Unanswered (n = 1)
Profession	Athletic Training (*n* = 33) Personal Training (*n* = 6) Physical Therapy (*n* = 19) Physical Therapy Aide (n = 3) Strength and Conditioning (*n* = 20) Other Athletic Training Student (*n* = 1) Lecturer of Exercise Science (n = 1) Occupational Therapy (*n* = 1) Semi-retired Consultant (n = 1)
Years in Profession	1–10 years (*n* = 50) 11–20 years (*n* = 18) 21–30 years (*n* = 1) 31 or more years (*n* = 1)

#### Education

Respondents suggested obtaining both formal education (*n* = 39, 55.7%) and self-education (*n* = 37, 52.9%) for their respective BFR/KAATSU devices. Of those who received formal training, 29 (74.4%) felt their training promoted a singular device, and 24 (61.5%) indicated their education was tailored toward a specific device. The majority (*n* = 58, 82.9%) felt that some sort of education should take place prior to BFR/KAATSU training implementation, while five felt education prior to implementation was not needed and an additional seven had no opinion on the matter.

#### Implementation

##### Barriers

Barriers were faced by 29 (41.4%) when trying to implement BFR/KAATSU training into practice. Barriers noted by those facing barriers included the cost of equipment (*n* = 20, 69%), lack of training (*n* = 10, 34.5%), doubts of effectiveness (*n* = 9, 31%), and a lack of clinical efficacy (*n* = 4, 13.8%). Other barriers noted were “concerns about medical complications (e.g., DVTs [Deep Vein Thrombosis])”, “concerns of medical staff”, “confidence in applying technique and having patient/client understand that BFR training is hard”, “lack of physician/surgeon buy-in”, “patient consent”, “patient fear”, “patients being willing to try it”, and “supervisor approval”.

##### Screening

Screening processes facilitated by respondents were comprised of medical screening forms including risk assessments and/or in person physical examinations (*n* = 27, 38.6%), both waiver/release forms and medical screening forms including risk assessments and/or in person physical examinations (*n* = 22, 31.4%), waivers/release forms (*n* = 1, 1.4%), and other screening processes (*n* = 2, 2.9%): “assure pt [patient] has no contraindication to BFR per a list and acquire consent from patient after describing treatment”, and “screening is done based off of recommendations of Owens Recovery Science”. Additionally, 57 (81.4%) respondents considered the psychosocial aspects related to BFR/KAATSU training. Eighteen (25.7%) did not conduct screening. Reasons suggested for a lack of screening were: “all participants are screened by medical department prior to contact with us”, “they are cleared by ATs[Athletic Trainers] for physical activity our requisites are met”, “initial health screening showed no signs of potential adverse interactions”, “we already know based on the medical history/chart if they are able to use this or not”, “communication with AT to determine if they are a good candidate for modality of BFR”, “we ask if they have history of blood clots”, “verbal consent”, “elite athletes”, “it is safe to use on the athletic population and patients I use it on”, “only self-use”, “I have only used on myself”, “use only on myself”, and “we just don’t have one outside of the one they sign for therapy”.

##### Application

Survey responses suggested the following methods to determine restrictive pressure: the use of comfort (i.e., “7/10” perceived tightness) (*n* = 13, 18.6%), limb circumference (*n* = 4, 5.7%), standard blood pressure (*n* = 5, 7.1%), doppler ultrasound (*n* = 11, 15.7%), or the device was set to determine restrictive pressure (*n* = 31, 44.3%). The remaining six responses (8.6%) provided other methods to determine restrictive pressures: “systolic pressure x 1.5”, “comfort and blood pressure”, “skin color, there should be a faint pulse, color should return to skin when pressed”, “capillary refill with progressive tightness based on both refill and feedback”, “device, will often lower pressure for first session”, and “not able to use any equipment”.

The majority (*n* = 67, 95.7%) believed personalizing restrictive pressure would reduce adverse effects, and multiple positions were used to determine restrictive pressure. Restrictive pressure determination was completed with the patient/client/athlete in a supine position (*n* = 33, 47.1%), seated position (*n* = 11, 15.7%), standing position (*n* = 9, 12.9%), and in an exercise dependent position (*n* = 17, 24.3%). For subsequent exercises, restrictive pressure was determined by the same measures as the initial assessment (*n* = 38, 54.3%), a different method from the initial method based on exercise position (*n* = 11, 15.7%), or no additional measurement of restriction pressure was made for subsequent exercises (*n* = 21, 30%). Workload was determined using heart rate (*n* = 5, 7.1%), percentage of 1 RM (*n* = 18, 25.7%), length of time under tension/load (*n* = 22, 31.4%), work to failure (*n* = 14, 20%), and other methods (*n* = 11, 15.7%). Other methods suggested were “using Delfi protocol, adding resistance if not worked to failure by end of protocol at next session”, “both %1 RM and length of time under tension”, “load and reps”, “low weight, high rep, 15–20 min”, “30/15/15/15”, “prescribed reps/sets from educational training”, “reps in deserve [sic], muscle fatigue scale”, “perceived exertion”, “RPE, by feel”, “muscle groups worked”, and “unknown”.

Blood flow restriction and KAATSU devices were applied for various lengths of time. Devices provided restriction for the duration of the workout (*n* = 24, 34.3%), devices were loosened or released between exercises (*n* = 29, 41.4%), devices were loosened or released between sets of an exercise (*n* = 10, 14.3%), or through other methods (*n* = 5, 7.1%); two individuals did not respond to the question. Other methods described by respondents were “as tolerated for prescribed exercise”, “client dependent-either intermittent or continuous”, “client dependent”, “provide restriction for duration up to 8 min max”, and “unknown”. The majority of respondents provided direct supervision to the patient/client/athlete while BFR/KAATSU training was being administered (*n* = 52, 74.3%). Additional respondents provided some supervision to the patient/client/athlete while BFR/KAATSU training was being administered (*n* = 14, 20%), while others provided no supervision to the patient/client/athlete while BFR/KAATSU training was being administered (*n* = 4, 5.7%).

Patients/clients/athletes received BFR/KAATSU training on the upper extremity (*n* = 4, 5.7%), lower extremity (*n* = 18, 25.7%), or both the upper extremity and lower extremities (*n* = 48, 68.6%). Activities for which BFR/KAATSU training were administered included strength training exercises (*n* = 47, 67.1%), aerobic exercise (*n* = 15, 21.4%), rehabilitation exercises (*n* = 57, 81.4%), and other activities (*n* = 5, 7.1%). Activities described were “active recovery”, “effects of BFR on sprint time”, “healing”, “I know PT’s [Physical Therapists] use it for rapid rehab after surgery”, and “recovery”. Specific forms of exercises performed with BFR/KAATSU can be seen in Table 3. Blood flow restriction and KAATSU training were administered: 1-2 sessions per week (*n* = 51, 72.9%), 3-4 sessions per week (*n* = 18, 25.7%), and 5-6 sessions per week (*n* = 1, 1.4%) but not 7 or more sessions per week (*n* = 0, 0%).

**TABLE 3:** Exercise employed with BFR/KAATSU training.

Types of Exercises Used with BFR/KAATSU Training	Number of Respondents Using This Form of Exercise (n = )
Single Joint Exercise	51
Single Joint Machine Based Exercise	37
Single Joint Free Weight Exercise	47
Multi Joint Exercise	57
Multi Joint Machine Based Exercise	32
Multi Joint Free Weight Exercise	49
Cycling	29
Walking	15
Jogging	10
Swimming	4
Rowing	1
Other	1
Recumbent stepper	—
Sport Specific	—

### Patient Demographics and Safety

The demographics of those for whom BFR/KAATSU training was applied can be seen in Table 4. Regarding safety, BFR/KAATSU training was administered on patients/clients/athletes with pathology by 16 (22.9%) respondents. Pathologies noted by respondents for which they have applied BFR/KAATSU training were hypertension, diabetes, obesity, EDS [Ehlers Danlos Syndrome], osteopenia, and unspecified cardiac conditions. Adverse effects from the administration of BFR/KAATSU training were seen by 15 (21.4%) respondents. Adverse effects seen can be seen in Table 5. Those who discontinued the use of BFR/KAATSU training did so for a variety of reasons presented in Table 6.

**TABLE 4:** Demographics.

Demographics of Those for which BFR was Applied to	
Gender	Male (*n* = 64, 91.4%) Female (*n* = 48, 68.57%) Gender Non-Conforming (*n* = 2, 2.9%) Transgender (*n* = 3, 4.3%) Gender unknown (*n* = 1, 1.4%)
Age (in years)	Under 20 (*n* = 45, 64.3%) 21–30 (*n* = 58, 82.9%) 31–40 (*n* = 31, 44.3%) 41–50 (*n* = 20, 28.6%) 51–60 (*n* = 12, 17.1%) 61 and older (*n* = 6, 8.6%)
Ethnicity	White (*n* = 61, 87.1%) Black, African American (*n* = 42, 60%) Asian (*n* = 16, 22.9%) Pacific Islander, Hawaiian (*n* = 10, 14.3%) Hispanic or Latino/a (*n* = 27, 38.6%) Native American or Alaskan Native (*n* = 11, 15.7%) Multi-racial (*n* = 1, 1.4%) Unknown ethnicity (*n* = 1, 1.4%)

**TABLE 5:** Adverse reactions described by respondents.

Described Demographics	Adverse Outcome	Other Noted Factors	Device Used	Screening Procedure
Elderly Age: 70s	Bruising, petechiae	—	Not enough info to determine device used; several devices indicated for current use	Waiver/Release and medical screening
Bodybuilding athlete Age: 40s	Bruising, petechiae	—	Not enough info to determine device used; several devices indicated for current use	Waiver/Release and medical screening
Male Age: 18–25	Giddy	—	Not enough info to determine device used; several devices indicated for current use	Medical screening
Caucasian female Age: 18–21	Lightheaded, increased body temperature	—	KELVI	Medical Screening
Athlete Age: college	Dizzy, lightheaded	Did not eat prior	Ace Bandage	No Screening
Caucasian female Age: mid 60s	Elevated heart rate, sweating, shortness of breath	—	Smart Cuffs	Waiver/Release and medical screening
Male Age: 40s	Increased pain with cuff occlusion	—	Delfi PTS-PBFR	Consent, ask contraindications
Unknown	Lightheadedness, muscle cramping	—	Delfi PTS-PBFR	Waiver/Release and medical screening
Hispanic Female Age: 21	Nausea, vomiting	—	Delfi PTS-PBFR	Waiver/Release and medical screening
Athletes High school, college Varied gender and race	Moderate cramping, lightheadedness, or did not tolerate sensation	—	Delfi PTS-PBFR	ORS specified

**TABLE 6:** Reasons respondents discontinued BFR/KAATSU training.

Non Safety Related	Safety Related	Other
Heavy load strength training is able to be performed consistently	Client was found to have developed a blood clot issue	Dangerous actions for people with poor health
It is used with our Physical Therapists and Sports Medicine Staffs in our settings. We have not incorporated in team/individual training, only utilize for personal use	Discomfort, Fatigue	Over time, a blood clot can develop that can lead to a fatal pulmonary embolism
I want to do it another way	Some tired, occasionally need a short rest	—
Money	Discomfort, had a patient who had fear of blood pressure cuffs but never told therapist, increased paraesthesia in the limb	—
N/A	Excessive pain, discomfort, or noticeable swelling	—
Progression to higher intensities due to rehab progress	Pain due to too much restriction	—
Time restrictions	Exercise pursor [sic] reflex symptoms	—
Time under pressure was reached	Extreme discomfort and loss of touch sensation	—
Wasn’t anything special	Failure or too uncomfortable for patient	—
When the patient reaches 15–20 min time of BFR cuff placed on leg or arm	Feeling much discomfort while exercising	—
Work reasons	Only use for 10–15 min	—
	If athlete complains of severe and unusual discomfort	—
	If the person cannot handle the pressure or repeatedly cannot hit target range	—
	Improper operation caused by bump	—
	Patient discomfort	—
	Patient discomfort, significant DOMS	—
	Perceived exertion gets too high or significant fatigue or muscle failure	—
	Prescreen, but if I find later that the person has a history of clotting I will discontinue	—
	Unable to tolerate the cuff, fatigue, inability to complete repetition range without severe compensatory patterns of movement	—
	Vomiting, lightheadedness	—

## Discussion

### Administration and Use of Various Forms of BFR/KAATSU Training Devices

The main finding of the research was the diversity in the selection and application of BFR. A variety of devices have been used in the facilitation of BFR/KAATSU training. The most common type of device applied was the inflatable device (43.5%, *n* = 47) followed by elastic tourniquet-based devices (19.4%, *n* = 21). Respondents reported equal use of KAATSU devices and knee wraps. Results of the current study were similar to a previous study by Patterson and Brandner (2018) where the use of inflatable devices, KAATSU devices, and knee wraps were comparable. One area that differed between the present study and Patterson and Bradner (2018) was the use of elastic tourniquet-based devices. While the present study found 19.4% of respondents (*n* = 108) have used an elastic tourniquet-based device, Patterson and Brandner (2018) found only 3.6% of respondents (*n* = 115) have used an elastic tourniquet-based device. Terminology used to describe the devices was based on Patterson and Brandner (2018) and may not reflect how respondents describe their devices, particularly tourniquet-based devices. Other terminology, including pneumatic tourniquet, has been used when describing tourniquet-based devices (McEwen et al., 2019; Patterson et al., 2019).

Among those administering BFR/KAATSU training at the time of the survey, respondants likewise employed a variety of devices.The most frequently applied device was still the inflatable device. This finding again mirrored Patterson and Brandner (2018) as handheld inflatable devices and automatic inflatable devices were reported as the most used devices.

At the time of the study, BFR/KAATSU training was being administered by those identifying as male/female genders across the country. Most predominantly, those administering BFR/KAATSU training were from a younger population (18–40 years old and practicing less than 20 years) and represented a variety of professions including athletic training, occupational therapy, physical therapy, personal training, and strength and conditioning. In previous survey-based research, authors have likewise noted administration by those of male and female genders (Nakajima et al., 2006; Yasuda et al., 2017; Patterson & Brandner, 2018; Mills et al., 2021), and administration by a younger demographic (Patterson & Brandner, 2018; Mills et al., 2021) across a variety of professions (Patterson & Brandner, 2018).

The present study also found 35.18% (*n* = 38) of individuals no longer administering BFR/KAATSU training. Minimal additional data was provided justifying discontinuation. Reasons that were cited included facility resources and facility policy on training prior to use of BFR/KAATSU training. While no additional literature could be found regarding those who have discontinued the use of BFR/KATTSU training, Mills et al. (2021) noted barriers among those who have never used BFR training included a lack of certification, training, and resources which mirrors concerns noted in the present study regarding facility policy and resources for BFR/KAATSU training use. Relative to discontinued use of BFR training, others have noted side effects or adverse reactions (Nakajima et al., 2006; Yasuda et al., 2017; Patterson and Brandner, 2018) could lead to temporary or permanent discontinuation of training. Side effects seen among those who were currently using devices can be found later in the discussion section.

Few researchers have assessed barriers implementing BFR/KAATSU training *via* survey research. In the present study, barriers were experienced by participants when implementing BFR/KAATSU training. The most frequently cited barrier was the cost of the apparatuses followed by a lack of training. While not assessed through the study, it can be noted that the most frequently cited devices (Table 1 and Table 7) have device specific training which can add to the potential cost for the user. Mills et al. (2021) found barriers to BFR implementation likewise included a lack of information, certification, and resources. In addition to the cost and training, some faced barriers on the effectiveness of BFR/KAATSU training, as well as concerns by overseeing medical practitioners or supervisors, and the patients/clients/athletes for which BFR/KAATSU training was being administered. Rolnick et al. (2021) identified screening safety, selecting an appropriate training pressure, device selection, and the influence of perceptual demands on compliance as barriers to BFR use. These factors likewise showed variability throughout the present study and may present barriers in the administration of BFR/KAATSU training.

**TABLE 7:** Actual device type.

Type of Device	Device Name
Tourniquet device	Delfi PTS-PBFR
Inflatable device	AirBands BFR Bands-Signature Series B Strong Fit Cuffs H + Cuffs MAD- UP Occlusion CuffSmart Cuffs The EDGE Restriction Systems VALD
KAATSU Training device	KAATSU Cycle 2.0 KAATSU Nano
Wraps	BFR Bands Koala Bands Rock Cuff
Other	3M ACE bandage Conmed/OBM HMKL KELVI (Cryo/Thermotherapy Device) LP Sports Protector Throwraft Original TD2401 (Personal Flotation Device)

The majority of respondents (82.9%, *n* = 58) believe training prior to BFR/KAATSU implementation should take place. While no additional information could be found regarding perceptions of BFR/KAATSU training implementation, respondents of this survey indicated training was necessitated by the BFR/KAATSU device company or the facilities where one is employed. Education received by respondents was both formal and self-facilitated but not all training promoted a singular device or was tailored toward a specific device. It is unknown how education was disseminated among the respondents of this survey.

Nearly three-quarters of respondents indicated conducting some sort of screening process and just over 80% considered the psychosocial aspects of BFR/KAATSU training. The most predominantly facilitated process was a medical screening or a medical screening and a waiver with the patient/client/athlete prior to use. Yasuda et al. (2017) also found most respondents performed interviews or assessments prior to application of KAATSU training either the first time or every time the device was applied. The present study also revealed 25.7% of respondents had no screening process. Upon further examination there were indications a screening process took place at some point. Comments on the open-ended question included reference to screenings by other departments and use of initial health screenings.

The same open-ended question suggested some screened on a limited basis or not at all. Those that assessed patients/clients/athletes on a limited basis suggested inquiring about blood clot history, while others asked for verbal consent. Also noted in the comments was the perception that no screening was needed when applying BFR/KAATSU training on those who were perceived as healthy. Patterson and Brander (2018) saw similar comments in which respondents felt there were no contraindications in populations of individuals who may be healthy, young, or athletic. In reviews of healthy populations, low intensity exercise with blood flow restriction has shown effects on hemodynamics within a normal spectrum (Neto et al., 2017) and improved strength gains and muscle mass greater than low intensity exercise alone (Slysz et al., 2016). Furthermore, stroke volume, blood pressure, heart rate, fibrinolytic potential, coagulation activity, and post occlusion blood flow responded the same as free flow high load resistance exercise in short term studies (Loenneke et al., 2011). Additionally, Patterson et al. (2019) suggested when applied and performed appropriately BFR should not produce muscle damage unless other susceptibility to adverse physiologic effects exist. For all populations, correct application and safety in training are important (Sato, 2005; Loenneke et al., 2011; Hughes et al., 2017; Patterson et al., 2019). Regardless, for those wanting to implement a screening tool, Kacin et al. (2015) created a screening questionnaire and Rolnick et al. (2021) proposed a funnel approach which can aid health professionals in determining if the treatment is appropriate.

The present study found 95.7% of those administering BFR/KAATSU training believed personalized restrictive pressure was needed to prevent adverse effects. There was variability in the procedures used to determine restrictive pressure. Techniques to determine restrictive pressure included the use of doppler ultrasounds, the device themselves, subjective rating scales and the use of capillary refill time. When administering BFR/KAATSU training, methods to obtain the pressure vary. For instance, the application of doppler ultrasound has shown reproducibility (Bezerra de Morais et al., 2017) and both the doppler ultrasound (Masri et al., 2016) and devices set to determine limb occlusion pressure (McEwan et al., 2019) have been advocated. For those unable to afford/operate doppler ultrasound, pulse oximeters have shown potential in determining occlusion pressure within the upper extremity (Zeng et al., 2019; Lima-Soares et al., 2020). Subjective rating scales can also be conducted with devices for which pressure cannot be determined through conventional means (Wilson et al., 2013); however, some have noted concerns with reliability of the use of the subjective rating scale to determine limb occlusion pressure (Bell et al., 2020). Additional procedures performed by respondents of the present study related to the use of skin color, pulse, and capillary refill time. Within the current study, 24.3% (*n* = 17) determined restrictive pressure in an exercise dependent position with 15.7% (*n* = 11) determining restrictive pressures for subsequent exercises using methods based on the exercise position. Sieljacks et al. (2018) and Hughes et al. (2018) demonstrated body position does influence arterial occlusion pressure in lower extremity exercise.

In this investigation, responses related to the administration of BFR/KAATSU training both matched (Nakajima et al., 2006; Patterson & Brandner, 2018; Patterson et al., 2019) and conflicted (Patterson & Brandner, 2018) with previous authors. Frequency of use was one similar area. In this study training methods were most applied 1–2 times per week (72.9%, *n* = 51) or 3-4 sessions per week (25.7%, *n* = 18). Authors have suggested BFR/KAATSU training was most administered one to three sessions per week (Nakajima et al., 2006), or one to two sessions and three to four sessions per week (Patterson & Brandner, 2018). Patterson et al. (2019) suggested administering BFR two to three times per week. Types of exercise employed also presented similarly between the current study and research from previous authors. Patterson and Brandner (2018) found cycling and walking were the most frequent aerobic exercises used with BFR which was reflected in the current study. Workload was one area which differed. Patterson and Brandner (2018) found most respondents determined workload using percentage of a one repetition maximum (1RM) with the following repetitions: 30 -15-15-15, or the use of repetitions to failure while the current study found length of time under tension/load was more frequently used than a percentage of 1RM or work for failure. Like Patterson and Brandner (2018), the results of this investigation indicate great variability in administration.

### Safety Topics Related to BFR/KAATSU Training With Various Devices

The second objective of the study was to explore safety related to the use of BFR/KAATSU training. The survey explored three areas related to safety. Safety topics addressed were the use of BFR/KAATSU training on individuals with pathology, adverse effects seen following device use, and reasons for discontinuing BFR/KAATSU training.

In the present study, 22.8% (*n* = 16) of respondents applied BFR/KAATSU training to those with pathology. Respondents indicated BFR/KAATSU training was most applied to individuals with obesity (37.5%, *n* = 6), hypertension (37.5%, *n* = 6), diabetes (25%, *n* = 4), and osteoporosis (12.5%, *n* = 1). Literature related to the use of BFR/KAATSU training with the four identified pathologies was limited. Nakajima et al. (2006) and Yasuda et al. (2017) have found practitioners using KAATSU training among those with obesity, hypertension, and diabetes. Bond et al. (2017) has assessed the effects of BFR on individuals who are both sedentary and obese finding increases in 1 RM and post occlusion blood flow. Nascimento et al. (2019) suggested greater understanding of blood flow restriction’s effect on coagulation would be beneficial for those at an increased risk of thrombi development including individuals with obesity, hypertension, and diabetes. Blood flow restriction has; however, shown positive hemodynamic effects (Loenneke et al., 2011; Neto et al., 2017; Yan et al., 2018; Nascimento et al., 2019) including among those with hypertension (Barili et al., 2018).

Specific to those who have diabetes, Kacin et al. (2015) indicated the potential risk of neurological injury caused by ischemia and nerve compression particularly among those with reduced peripheral nerve function. Few studies have explored the effects of administering BFR/KAATSU training on those with osteoporosis. Silva et al. (2015) found a small sample of women with osteoporosis were able to improve maximal dynamic strength on knee extension exercise and Yasuda et al. (2017) found practitioners using KAATSU training among individuals with osteoporosis.

For those uncertain how BFR/KAATSU training responds within a population or those with pathologies for which the efficacy of BFR/KAATSU training has not been ascertained, including the pathologies noted by respondents of the present study, some additional recommendations have been made. Nascimento et al. (2019) proposed an alternative exercise regime for resistance training using 50% of the 1 RM. In addition, Kacin et al. (2015) developed a screening tool and Rolnick et al. (2021) a funnel which may help in determining whether to administer BFR/KAATSU training. Finally, Patterson et al. (2018) suggested the use of clinical prediction rules to assess for additional risk particularly for venous thromboembolism.

Adverse effects were seen by those applying devices marketed for BFR/KAATSU training as well as those applying devices not marketed for BFR/KAATSU training. Details about adverse reactions can be seen in Table 5. With the exception of one adverse effect where prior food consumption was called into question, it is unknown if other personal factors influenced the adverse reaction. The adverse effects described in this investigation matched common reactions presented by other authors (Nakajima et al., 2006; Yasuda et al., 2017).

Individuals discontinued the use of BFR/KAATSU training due to changes to training, facility concerns, monetary issues, as well as safety. Reasons for discontinuation of BFR/KAATSU training (Table 6) directly related to side effects (e.g. lightheadedness and pain) were similar to side effects reported previously (Nakajima et al., 2006; Yasuda et al., 2017; Patterson and Brandner, 2018). Nascimento et al. (2019) recommended further research to quantify side effects to develop clearer parameters for use particularly among patients/clients/athletes who may have pathology or who may be older. Furthermore, quantifying a side effect versus an adverse reaction may limit ambiguity seen in the present study.

## Limitations

There were limitations in the current study. The survey did not go through content validation nor were content validation coefficient statistical analysis completed following the development of the survey. A single test pilot was completed; however, additional revisions and analysis could have been completed to ensure its validity. Additionally, the survey was long at 37 questions taking an average of nearly 11 min to complete. Future investigations should explore survey constructs including verbiage for greater clarity.

The majority of the survey were selection-based questions. The questions potentially prevented respondents from elaborating or required a best fit answer which may not reflect what was actually being done. Participants were however, given the opportunity to provide written responses on several constructs. The written work likewise posed limitations. Some of the written work presented incomplete thoughts and typographical errors limiting the ability to interpret what was written.

Finally, COVID-19 pandemic was still taking place at the time of the survey. While there was some return daily life; it is unknown if the constraints of the pandemic precluded some participants from participating as he/she/they may have been unable to used BFR/KAATSU training based on their particular circumstances.

## Conclusion

Overall, the study demonstrated diversity in the use of blood flow restriction. Devices used by participants varied in style and brand including those marketed and not marketed for BFR/KAATSU specific use. Barriers were seen by some when trying to implement BFR/KAATSU training. Formal training, self-training, or a combination of both were completed by most study participants. Many noted the inclusion of some sort of screening process prior to administering BFR/KAATSU training. The methodologies used to administer BFR/KAATSU training were vast. Adverse effects were seen by participants and BFR/KAATSU training was administered to those with pathology. Finally, discontinuation of BFR/KAATSU training occurred for reasons directly related to BFR/KAATSU training application and non-device related factors.

## Data Availability

The original contributions presented in the study are included in the article/Supplementary Material, further inquiries can be directed to the corresponding author.
