# Supplementary material for: Current Trends in Blood Flow Restriction
Source: Front Physiol. 2022 Jul 6;13:882472. doi: 10.3389/fphys.2022.882472 (PMC9298746; doi:10.3389/fphys.2022.882472)
Supplement: Supplementary file 1 [file DataSheet1.PDF]

frontiers

Supplementary Material

1 Supplementary Data

Supplementary Material should be uploaded separately on submission. Please include any supplementary data, figures and/or tables. All supplementary files are deposited to FigShare for permanent storage and receive a DOI.

Supplementary material is not typeset so please ensure that all information is clearly presented, the appropriate caption is included in the file and not in the manuscript, and that the style conforms to the rest of the article. To avoid discrepancies between the published article and the supplementary material, please do not add the title, author list, affiliations or correspondence in the supplementary files.

2 Supplementary Figures and Tables

For more information on Supplementary Material and for details on the different file types accepted, please see here. Figures, tables, and images will be published under a Creative Commons CC-BY licence and permission must be obtained for use of copyrighted material from other sources (including re-published/adapted/modified/partial figures and images from the internet). It is the responsibility of the authors to acquire the licenses, to follow any citation instructions requested by third-party rights holders, and cover any supplementary charges.

Survey Questions

Current Use of Blood Flow Restriction

Product Use: The following set of questions relate to the blood flow restriction/KAATSU training products which you have or are currently using.

Which of the following types of devices have you used previously to apply restriction? (please select all that apply.)

- ▢ Elastic tourniquet (1)
- ▢ Inflatable device (2)
- ▢ KAATSU training device (3)
- ▢ Knee wraps (4)
- ▢ Other-Please specify (5) ____

What device are you currently using to apply restriction?

o Elastic tourniquet. Please identify brand/style of device. (1) ____

o Inflatable device. Please identify brand/style of device. (2) ____

o KAATSU training device. Please identify style of device. (3) ____

o Knee wraps. Please identify brand/style of wrap. (4) ____

o Other- Please specify (5) ____

o I do know what type of device I am using to apply restriction. Please identify brand/style of device. (7) ____

o I am not currently using blood flow restriction/KAATSU training. Please indicate why blood flow restriction/KAATSU training is no longer being used by you for patient, client, or athlete care. (6) ____

Skip To: End of Survey If What device are you currently using to apply restriction? = I am not currently using blood flow restriction/KAATSU training. Please indicate why blood flow restriction/KAATSU training is no longer being used by you for patient, client, or athlete care.

Years device used Please answer all remaining questions as they apply to the device you are currently using.

How many years have you used this device?

o 0-1 years (1)

o 2-5 years (2)

o 6-10 years (3)

o 11-15 years (4)

o 16-20 years (5)

o 20+ years (6)

For what reasons are you using this device? (please select all that apply)

▢ Strength training (1)

▢ Rehabilitation (2)

▢ Aerobic training (3)

▢ Other-Please specify (4) ____

What training did you complete prior to utilizing this blood flow restriction/KAATSU training device? (please select all that apply).

▢ Self educated (1)

▢ Formal course work (2)

Display This Question:

If What training did you complete prior to utilizing this blood flow restriction/KAATSU training dev... = Formal course work

In the formal course work you received, was the education paired with promoting a specific device?

o Yes (1)

o No (2)

Display This Question:

If In the formal course work you received, was the education paired with promoting a specific device... = Yes

Do you feel that the education you received was tailored towards the use of that device?

o Yes (1)

o No (2)

Do you believe there should be formal education on blood flow restriction/KAATSU training before being allowed to use it with patients/clients/athletes?

o Yes (4)

o No (5)

o No opinion (6)

Did you face any barriers implementing blood flow restriction/KAATSU training into practice?

o Yes (1)

o No (2)

Display This Question:

If Did you face any barriers implementing blood flow restriction/KAATSU training into practice? = Yes

What barriers did you face when integrating blood flow restriction/KAATSU training into practice? (please select all that apply)

- ☐ Lack of training (1)
- ☐ Equipment cost (2)
- ☐ Doubts of effectiveness (3)
- ☐ Lack of clinical efficacy (4)
- ☐ Other-Please explain (5) ____

Current Uses: The following set of questions relate to your current use of blood flow restriction/KAATSU training.

Which body parts are you applying exercise with blood flow restriction/KAATSU training?

- o Upper Extremity (1)
- o Lower Extremity (2)
- o Both Upper and Lower Extremity (3)

Which types of exercises are being performed with blood flow restriction/KAATSU training? (please select all that apply)

- ☐ Single joint exercise (1)
- ☐ Single joint machine based exercise (2)
- ☐ Single joint free weight based exercise (3)
- ☐ Multi joint exercise (4)
- ☐ Multi joint machine based exercise (5)
- ☐ Multi joint free weight based exercise (6)
- ☐ Cycling (7)
- ☐ Jogging (8)
- ☐ Swimming (9)
- ☐ Rowing (10)

☐ Walking (11)

☐ Other types of exercise-Please specify (12) ____

On average, how often will you have a patient/client/athlete complete exercises with blood flow restriction/KAATSU training?

o 1-2 sessions per week (1)

o 3-4 sessions per week (2)

o 5-6 sessions per week (3)

o 7+ sessions per week (4)

What method are you using to determine the restrictive pressure of your blood flow restriction/KAATSU training device?

o Comfort (ie “7/10” perceived tightness) (1)

o Limb circumference (2)

o Standard blood pressure (3)

o Doppler ultrasound (4)

o The device is set to determine restrictive pressure (5)

o Other-Please specify (6) ____

What position is the patient/client/athlete when initially taking personalize restrictive pressure?

o Supine (1)

o Seated (2)

o Standing (3)

o Exercise dependent (4)

Do you use the ${position restr pr/ChoiceGroup/SelectedChoices} position to determine restrictive pressure for subsequent exercises which vary in position (ie. supine straight leg raise, seated leg extensions, squats)?

o Yes, I repeat the same method to determine restrictive pressure on subsequent exercises regardless of exercise position (1)

o No, I use different methods to determine restrictive pressure on subsequent exercises which vary in exercise position (3)

o I do not retake personalized restrictive pressure on subsequent exercises (2)

Do you feel that personalizing the pressure to the individual reduces the risk of adverse events during blood flow restriction/KAATSU training exercise?

o Yes (4)

o No (5)

When applying blood flow restriction/KAATSU training what are you using to consider work load?

o Heart rate (1)

o Percentage of 1 RM (2)

o Length of time under tension/load (3)

o Work to failure (4)

o Other-Please describe (5) ____

Generally, how long is the device providing restriction to a patient/client/athlete completing exercise?

o The device provides restriction for the duration of the workout (1)

o The device is loosened or released between exercises (continuous application) (2)

o The device is loosened or released between sets of an exercise (intermittent application) (3)

o Other-Please describe (4) ____

Safety: The following set of questions relate to safety during blood flow restriction/KAATSU training with your current device.

What systematic screening process is used to determine eligibility of blood flow restriction/KAATSU training in patients/clients/athletes?

o Waiver/Release forms (4)

o Medical screening forms including risk assessments and or in person physical examinations (5)

o Both waiver/release forms and medical screening forms including risk assessments and or in person physical examinations (8)

o Other-Please describe systemic screening process being used. (6) ____

o No screening process is used to determine eligibility. Please explain the reason for no screening process. (7) ____

Do you consider the psychosocial aspects surrounding the application of blood flow restriction/KAATSU training (eg. attitude/beliefs about exercise, the degree of exercise-induced discomfort, patient/client/athlete adherence) when determining its use with a patient/client/athlete?

o Yes (4)

o No (5)

How much supervision is provided when a patient/client/athlete is completing exercise with blood flow restriction/KAATSU training?

o Patient/client/athlete performing exercises with blood flow restriction/KAATSU training has no supervision for the duration of blood flow restriction/KAATSU training use (1)

o Patient/client/athlete performing exercises with blood flow restriction/KAATSU training has some supervision for the duration of blood flow restriction/KAATSU training use (2)

o Patient/client/athlete performing exercises with blood flow restriction/KAATSU training is directly supervised for the duration of blood flow restriction/KAATSU training use (3)

Have you performed blood flow restriction/KAATSU training in individuals with medical comorbidities (ie diabetes, hypertension, obesity etc)?

o Yes-Please indicated comorbidities seen. (1)
____

o No (2)

Have you seen any adverse effects in patients/clients/athletes either during a session or following a session using your current device?

o Yes (1)

o No (2)

Display This Question:

If Have you seen any adverse effects in patients/clients/athletes either during a session or followi... = Yes

What adverse effects have you seen in patients/clients/athletes completing exercise with restricted blood flow, either during a session or following a session using your current device? (If possible please include gender, race, approximate age range, and adverse effect).

____

____

____

____

____

For what reasons have you discontinued use of exercise with blood flow restriction/KAATSU training while exercise is being performed?

____

____

____

____

____

Patient/Client/Athlete Demographics: The following questions relate to the demographics of the patients/clients/athletes for which blood flow restriction/KAATSU training has been applied.

Identify the genders of the patients/clients/athletes completing exercise with blood flow restriction/KAATSU training? (please select all that apply)

- ▢ Male (1)
- ▢ Female (2)
- ▢ Gender Nonconforming (3)
- ▢ Transgender (4)
- ▢ Unknown (5)
- ▢ Other-Please identify (6) ____

What were the ethnicities (races) of the patients/clients/athletes completing exercise with blood flow restriction/KAATSU training? (Select all that apply)

- ▢ White (1)
- ▢ Black or African American (2)
- ▢ American Indian or Alaskan Native (3)

☐ Hispanic or Latino/a (4)

☐ Asian (5)

☐ Native Hawaiian or Pacific Islander (6)

☐ Other-Please identify (8) ____

What were the age groups of the patients/clients/athletes completing exercise with blood flow restriction/KAATSU training?

☐ <21 years old(1)

☐ 21-30 years old (2)

☐ 31-40 years old (3)

☐ 41-50 years old (4)

☐ 51-60 years old (5)

☐ 61+ years old (6)

Your Demographics: The following questions relate to your demographics.

Please select the gender you identify.

o Male (1)

o Female (2)

o Gender Nonconforming (3)

o Transgender (4)

o Other-Please identify (5) ____

Please select your age group.

o 18-30 years old (1)

o 31-40 years old (2)

o 41-50 years old (3)

o 51-60 years old (4)

o 61+ years old (5)

Please identify your ethnicity (race). Select all that apply

- ▢ White (1)
- ▢ Black or African American (2)
- ▢ American Indian or Alaska Native (3)
- ▢ Asian (4)
- ▢ Native Hawaiian or Pacific Islander (5)
- ▢ Hispanic or Latino/a (6)
- ▢ Other-Please identify (7) ____

Which region of the United States do you currently reside?

- o Northeast (1)
- o Southeast (2)
- o Midwest (3)
- o West (4)
- o Northwest (5)
- o Southwest (6)

What is your current profession? (Select all that apply)

- ▢ Athletic Trainer (41)
- ▢ Chiropractor (42)
- ▢ Physical Therapist (43)
- ▢ Physical Therapist Assistant (48)
- ▢ Personal Trainer (44)
- ▢ Strength and Conditioning Specialist (45)
- ▢ Other-Please identify (47) ____

How many years have you been in your current profession?

- o 0-10 years (1)
- o 11-20 years (2)

o 21-30 years (3)

o 31+ years (4)
